# EGFR is a pivotal regulator of thrombin-mediated inflammation in primary human nucleus pulposus culture

**DOI:** 10.1038/s41598-017-09122-3

**Published:** 2017-08-17

**Authors:** Bor-Ren Huang, Tzu-Sheng Chen, Da-Tian Bau, I-Chen Chuang, Cheng-Fang Tsai, Pei-Chun Chang, Dah-Yuu Lu

**Affiliations:** 1 Graduate Institute of Clinical Medical Science, China Medical University, Taichung, Taiwan; 2 Department of Neurosurgery, Taichung Tzu Chi Hospital, Buddhist Tzu Chi Medical Foundation, Taichung, Taiwan; 3 School of Medicine, Tzu Chi University, Hualien, Taiwan; 4 Department of Pathology, Taichung Tzu Chi Hospital, Buddhist Tzu Chi Medical Foundation, Taichung, Taiwan; 5 Department of Pharmacology, School of Medicine, China Medical University, Taichung, Taiwan; 6 Department of Biotechnology, Asia University, Taichung, Taiwan; 7 Department of Bioinformatics, Asia University, Taichung, Taiwan; 8 Department of Photonics and Communication Engineering, Asia University, Taichung, Taiwan

## Abstract

We found that the coagulation and cytokine pathways were important mechanisms involve in the degeneration of intervertebral discs (IVD) using a microarray approach to analyze gene expression in different grades of specimens. Furthermore, using a cytokine/chemokine array, a significant increase in CXCL8 expression was observed in human nucleus pulposus (NP) cells after thrombin treatment. The enhancement of CXCL8 expression by thrombin was activated by the PAR1 receptor. Importantly, analysis of degenerated human NP tissue samples showed that EGFR expression positively correlated with the grade of tissue degeneration. In NP cells, thrombin caused an increase in phosphorylation of the EGFR at the Tyr1068, and treatment with the pharmacological EGFR inhibitor, AG1473 effectively blocked thrombin-enhanced CXCL8 production. Surprisingly, inhibition of STAT3 for 24 h decreased expression of EGFR. Treatment with thrombin also increased Akt and GSK3α/β activation; this activation was also blocked by EGFR inhibitor. Although c-Src, ERK, and FAK were activated by thrombin, only c-Src and ERK were involved in the STAT3/CXCL8 induction. Our findings indicate that stimulation of an inflammatory response in NP cells by thrombin is part of a specific pathophysiology that modulates the EGFR activation through activation of Src/ERK/STAT3 signaling.

## Introduction

Degeneration of the intervertebral discs (IVD) involves structural disruption and cell-mediated changes in its composition, and is associated with low back pain^1–3^. However, a specific pharmacological treatment for degenerative disc disease is still needed^4^. Disc degeneration results from an imbalance between the degradation and synthesis of extracellular matrix components and an overall shift toward fibrotic matrix synthesis by chondrocytes residing in the gel-like nucleus pulposus (NP)^5^. The human IVD can be macroscopically separated into two main components, comprising the NP and the annulus fibrosus (AF). Structural disruption of the IVD leads to herniation of NP tissue, which is often followed by inflammatory responses, characterized by infiltration of immune cells into the tissue^6^. Degenerated discs have significant neovascular ingrowth between the NP cells^7^ which also causes an inflammatory reaction, along with leukotaxis and increased vascular permeability^8^. Importantly, during disc degeneration, NP cells produce molecules associated with nerve growth and angiogenesis that cause immune cells infiltration^9, 10^. Moreover, previous studies have reported that the regulatory effect of inflammatory cytokines in NP cells is associated with degenerative disc disease^11, 12^.

Thrombin, a multifunctional enzyme, can modulate both hemostasis and coagulation^13^ and can activate intracellular signaling pathways by interacting with protease-activated receptors (PARs). The three PAR family members, PAR-1, PAR-3, and PAR-4, are cleaved by thrombin, and PARs have been implicated in the development of acute and chronic inflammatory responses^14^. Both thrombin^15^, and its activator^16^, are expressed in the CNS, and thrombin dynamically regulates cell growth, development, and response to injury in the central and peripheral nervous systems^17^. Low concentrations of thrombin induce tolerance against various insults while high concentrations of thrombin induce apoptotic cell death^18, 19^. In various types of nervous system injuries and diseases, secretion of thrombin is associated with cell degeneration, which involves numerous cytokines and chemokines^17, 20^. A clinical study has shown that levels of prothrombin, the thrombin precursor, increase with age^21^. It has also been reported that spinal cord parenchymal accumulation of thrombin results in blood-spinal cord barrier breakdown in amyotrophic lateral sclerosis patients^22^. Spinal motor neurons express the thrombin receptor PAR-1, and activation of this receptor induces neuronal cell degeneration and death^23, 24^. Furthermore, activation of the PAR-1 receptor in the lamina I and II regions of the spinal cord modulates neuropathic pain^25^.

Chemokines constitute of a group of pro-inflammatory cytokines with potent chemotaxis activity that can target specific inflammatory cells^26^ and recruit them from the blood into tissues^27^, and in this way drive the chronic inflammatory process. Several cytokines and chemokines are involved in the development of inflammation and are responsible for the pathology of IVD degeneration^28^. CXCL8 has been considered to participate in the pathomechanism of nerve root inflammation in lumbar disc herniations and to be associated with development of radicular pain by back extension^29^. A recent study has reported that patients with lumbar radicular pain due to disc herniation have increased serum levels of CXCL8^30^. Substance P, a neurotransmitter that acts as an inflammatory mediator, stimulates CXCL8 upregulation in NP cells^31^. Importantly, the concentration of CXCL8 has been found to be significantly higher in patients with spinal cord injury than in healthy controls, and was negatively correlated with the duration of spinal cord injury^32^. The increased level of CXCL8 within the nucleus pulposus has been reported to be related to neovascularization in patients with discogenic pain^33^, and a CXCL8 inhibitor has been suggested as a promising therapeutic agent for disc herniation that causes chronic radicular neuropathic pain^34^. Importantly, the protein expression of CXCL8 by NP cells is significantly increased concordant with the increasing severity of degenerative tissue changes in human NP^35^. Gene expression analysis has also indicated that CXCL8 expression was greater in IVDs with immune cell infiltration compared to that in nondegenerative IVD^35^. Although the roles of chemokines in immune cell chemotaxis have been described in detail, the regulation of CXCL8 during disc degeneration is still not clear.

In this study, immunohistochemistry revealed that higher levels of the EGFR were found in NP cells and were associated with higher clinical pathological stages of IVD in degenerative patients. Our results also show that EGFR-activated Akt/GSK3 signaling promotes CXCL8 expression. Stimulation by thrombin upregulates EGFR-dependent CXCL8 expression through the Src/ERK/STAT3 signaling pathways in NP cells, which may contribute to immune cell recruitment and disc degeneration.

## Materials and Methods

All methods were performed in accordance with the relevant guidelines and regulations.

### Reagents

Human recombinant thrombin was purchased from PeproTech (Rocky Hill, NJ, USA). Cytokine/chemokine array membranes were purchased from R&D Systems (Minneapolis, MN, USA). Dulbecco’s modified Eagle’s medium (DMEM), and fetal bovine serum (FBS) were purchased from Gibco-BRL (Invitrogen Life Technologies, Carlsbad, CA, USA). Secondary antibodies, primary antibodies against phospho-c-Src, phospho-Akt, phospho-ERK1/2, c-Src, Akt, ERK2, FAK, EGFR, and β-actin were purchased from Santa Cruz Biotechnology (Santa Cruz, CA, USA). Primary antibodies against phospho-FAK, phospho-GSK3α/β, GSK3α/β, and phospho-EGFR were purchased from Cell Signaling and Neuroscience (Danvers, MA, USA). Pre-immune rabbit IgG was purchased from Abcam (Cambridge, MA, USA). LY294002, PP2, SB216763, PPACK and diaminobenzene were obtained from Sigma-Aldrich (St. Louis, MO, USA). U0126, WP631, and AG1478 were obtained from Tocris Bioscience (Ellisville, MO, USA). AG490, the JAK inhibitor 1, PF573228, and the STAT3 inhibitor were purchased from Calbiochem (La Jolla, CA, USA). The avidin-biotin complex (ABC) standard kit was purchased from Vector Laboratories (Burlingame, CA, USA).

### Human tissue collection and grading

**Figure 1:**
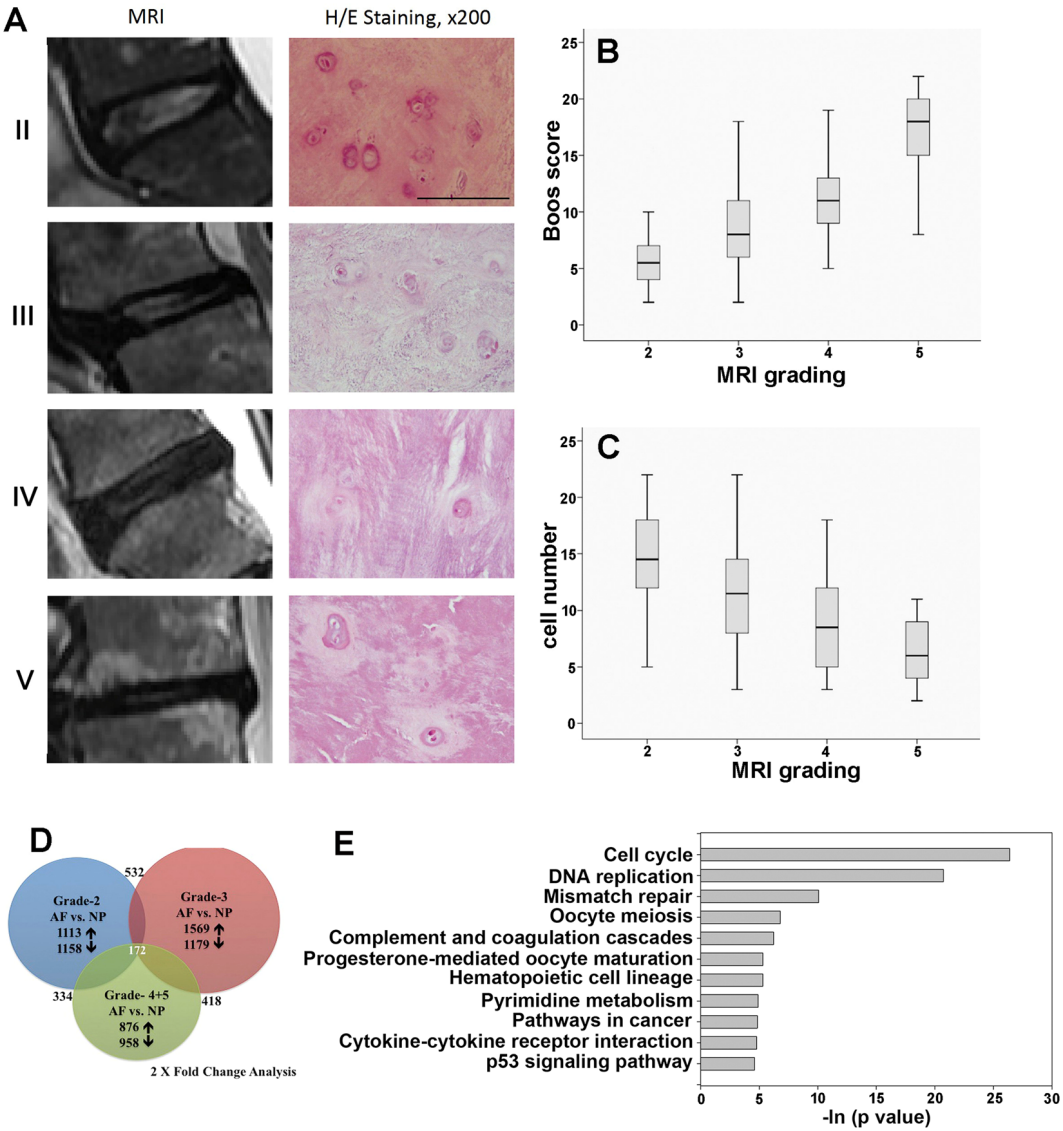
Histology of intervertebral disc in discectomy patients. (**A**) Typical MRI images of patients with Pfirrmann grades 2 to 5. NP cell density and extracellular matrix decreased with the IVD degenerative process, and round cartilage-like cells are replaced by fibroblasts (right column H/E, 200×). Scale = 100 μm. (**B**) Comparison of the Boos score with the MRI grade, *p* < 0.001 by one-way ANOVA method. (**C**) Comparison of cell number with MRI grade, cell number was counted in one field at 200 × magnification under a microscope, *p* < 0.001 calculated by one-way ANOVA. (**D**) Proportional Venn diagram illustrating the genes significantly altered by AF and NP at different grades. The number of genes (fold change threshold > 2) unique to each comparison that were increased or decreased are listed next to arrows indicating the direction of change. The Venn diagram reveals a core of 172 genes that were changed in AF compared with NP across all grades. The large number of unique gene expression changes in each condition illustrates that the gene expression is altered by the clinical stage of the patient (blue = grade 2, red = grade 3, green = grade 4 & 5). (**E**) Functional analysis of the differentially regulated genes was carried out using Kyoto Encyclopedia of Genes and Genomes (KEGG) database (http://www.kegg.jp/ or http://www.genome.jp/kegg/). The functional annotation clusters are shown as Pathways that had a p value < 0.01.

Human IVD samples (Table 1) were obtained during spinal surgery with approval of the Taichung Tzu-Chi hospital ethics committee (REC102-14 and REC103-55). IVD degenerative specimens were obtained from patients who underwent surgical discectomy for cervical, lumbar, or sacral discs (Table 2). These IVD degenerative specimens were obtained as surgical waste from patients who underwent surgical discectomy for lumbar discs. All informed consents were obtained from patients themselves, or their family members, prior to tissue collection. Assessment of the disease state was performed using Pfirrmann grading^36^. This scheme uses a T2-weighted magnetic resonance imaging with image analysis by three independent observers. The Pfirrmann grade covers the spectrum from a healthy disc (Grade I) to discs with advanced degeneration (Grade V, the most advanced stage of degeneration). The patients’ age, gender, spinal level, NP tissue grade, and surgical diagnosis of contained or non-contained disc, are listed in Tables 1 and 2. Additionally, we assessed the disc degenerative condition using another scoring system based on pathologic results^37^. The Boos score analyzes the degenerative condition based on chondrocyte proliferation, mucus degeneration, cell death, tear and cleft formation, and granular changes and is scored from 0 to 22. We also compared the Pfirrmann grade from the MRI analysis with the pathological Boos score and they were found to be closely correlated (Fig. 1B).

**Table 1:** Clinical characteristics of the study population.

	Grade I/II	Grade III	Grade IV	Grade V	Total
Spinal level					
C3-C4		4	1		5
C4-C5	3	1	1	3	8
C5-C6	3	11	3		17
C6-C7	1	2	1		4
C7-T1			1		1
L2-L3		1	1		2
L3-L4		3	4		7
L4-L5		12	9	5	26
L5-S1	3	6	7	1	17

**Table 2:** Spinal levels and clinical pathology of the study population.

	Grade I/II	Grade III	Grade IV	Grade V	Total
Age	10	40	28	9	87
20–35	1	5			6
36–45	2	8	3		13
46–55	4	10	4	2	20
56–65	3	11	14	1	29
>66		6	7	2	19
Gender					
Male	4	23	10	7	44
Female	6	17	18	2	43
Disc pathology					
Contained	6	22	22	8	58
Non-contained	4	18	5	1	28

### Isolation and cell culture of NP and AF from tissue specimens

Human NP and AF cells were isolated used a non-enzymatic method that were modified from previous studies^38, 39^, which did not cause damage to cell surface receptors and preserved its phenotypes^39–41^. Human disc tissue (minimally degenerative) was obtained from patients undergone cervical spinal surgeries as per an approved protocol at Taichung Tzu Chi Hospital, Buddhist Tzuchi Medical Foundation. Tissues were anonymized, with only the patient age, gender, and grade recorded. Disc tissues were rinsed with PBS and grossly separated into AF and nucleus NP. Any other non-disc materials such as endplate bone or cartilage in the surgical sample were discarded prior to tissue incubation. Separated AF and NP tissues were further washed three times with a washing medium [DMEM basal medium with 100 μg/mL kanamycin (Sigma, St. Louis, USA) and 165 μg/mL gentamycin (Gibco, Grand Island, NY, USA), and 1.25 μg/mL fungizone (Gibco)] and cut into small pieces (the average size of the tissue explant was 1–2 mm^3^ for AF, and 3–5 mm^3^ for NP). NP and AF cells were maintained in DMEM/F12 supplemented with antibiotics and 10% FBS. The culture medium was changed every two days. Once cells had migrated out of the tissue and had been expanded for three to four weeks (to about 80% of confluence), the tissue explants were transferred to a new flask for another round of incubation, with the remaining cells in the original flask being ready for harvesting. In general, incubated tissues could be transferred up to ten times or until cell migration ceased.

### Patients sample microarray

We selected sixteen patients (the clinical-pathological data are summarized in Table 3) with IVD degenerative specimens and performed a cDNA microarray. RNA labeling and hybridization were performed using a kit from Welgene Biotech Co., Ltd (Welgene Biotech Co., Ltd., Taipei, Taiwan) according to the manufacturer’s instructions. Briefly, 0.2 μg of total RNA was amplified using a Low Input Quick-Amp Labeling kit (Agilent Technologies) and labeled with Cy3 (CyDye, Agilent Technologies) during the *in vitro* transcription process. Cy3-labeled cRNA (0.6 μg) was fragmented to an average size of about 50–100 nucleotides by incubation with fragmentation buffer at 60 °C for 30 min. The fragmented labeled cRNA was then pooled and hybridized to an Agilent SurePrint G3 Human V2 GE 8 × 60 K Microarray (Agilent Technologies, USA) at 65 °C for 17 h. After washing and then drying using a nitrogen gun, microarrays were scanned with an Agilent microarray scanner (Agilent Technologies) at 535 nm to detect the Cy3 signal. Scanned images were analyzed using the Feature extraction 10.5.1.1 software (Agilent Technologies). Image analysis and normalization software was used to quantify the signal and background intensity for each feature.

**Table 3:** Clinical pathology and data for tissue samples used in the microarray analysis.

Pfirrmann grade	Grade II	Grade III	Grade IV, V
Age	52	54	74
Females/males	F	M	F
Spinal level	L4-L5	L4-L5	L4-L5
Specimens	NP, AF	NP, AF	NP, AF
Age		44	71
Females/males		M	F
Spinal level		L4-L5	L4-L5
Specimens		NP, AF	NP, AF
Age		36	84
Females/males		M	M
Spinal level		L4-L5	L3-L5
Specimens		NP, AF	NP, AF
Age		51	
Females/males		F	
Spinal level		C6-C7	
Specimens		NP, AF	

### Non-degenerative human NP cells

Healthy human NP cells from the human intervertebral disc were purchased from Sciencell Research Laboratories (Cat# 4800, Carlsbad, CA, USA). Cells were cultured in Nucleus Pulposus Cell Medium with supplements (NPCM, Sciencell, Cat.#4801) on poly-L-lysine coated tissue culture dishes. The media was changed every two days and cells were passaged when they were over 90% confluent. Cells were cultured in medium supplemented with 10% heat-inactivated FBS, 100 U/mL penicillin, and 100 mg/mL streptomycin at 37 °C, and incubated in a humidified atmosphere consisting of 5% CO_2_ and 95% air.

### Immunohistochemical Staining

The protocol for immunohistochemistry was slightly modified from our previous report^42^. Briefly, tissue sections measuring 4 μm were dewaxed and rehydrated, and endogenous peroxidases were quenched and, following heat-mediated antigen retrieval, blocked in normal goat serum. Sections were incubated overnight at 4 °C with primary antibody against EGFR. Pre-immune rabbit IgG was used as a negative control. Binding was detected using a biotinylated secondary antibody and an ABC standard kit. Visualization was performed using 0.05% diaminobenzene. After slices were stained with hematoxylin staining for counter staining, slices were mounted, and visualized with Zeiss microscope. Slides were evaluated independently by a pathologist who was blinded to the patients’ outcomes.

### Cytokine/chemokine array

Supernatants collected from NP cells were then removed and clarified with a 20,000 *g* spin (10 s at 4 °C), and applied to each of two separate cytokine array membranes. Membranes were processed following the manufacturer’s instructions. Chemi-luminescence was detected using LAS-3000 film (Fuji; Tokyo, Japan). Manual background subtraction was performed prior to quantitation, and “fold change” was calculated as the difference between the average values for both cytokine spots on thrombin-treated versus untreated arrays.

### Western blot analysis

The western blotting procedure has been previously described^43^. Briefly, cells were lysed with immunoprecipitation assay buffer on ice. Protein samples were separated by sodium dodecyl sulfate-polyacrylamide gel electrophoresis, and transferred to polyvinylidene difluoride membranes. The membranes were blocked with 5% nonfat milk and then probed with appropriate primary antibody. The blots were visualized using enhanced chemiluminescence and LAS-3000 film. The blots were subsequently stripped by incubation in a stripping buffer and re-probed for β-actin as a loading control. Quantitative data were obtained using a densitometer and ImageJ software.

### ELISA Assays

Production of CXCL8 in the culture supernatant was measured using a commercial kit (R&D Systems) according to the manufacturer’s instructions. The absorbance was determined using a microplate reader.

### RNA extraction and Quantitative real time-PCR

Quantitative real-time PCR was performed using SYBR-green detection of PCR products in real time using the 96-well StepOne Plus Real Time System (Applied Biosystems, Foster City, CA, USA). The threshold was set within the linear phase of the target gene amplification to calculate the cycle number at which point the transcript was detected (denoted as CT). Primers used for quantitative PCR were as follows:

CXCL8: 5′-AGGTGCAGTTTTGCCAAGGA-3′ and 5′-TTTCTGTGTTGGCGCAGTGT-3′;

PAR1: 5′-CACGGCAGATGTGCTGTTTGTG-3′ and 5′-AGAGGGACTGCATGGGATACAC-3′;

ICAM1: 5′-CCCCCCGGTATGAGATTGT-3′ and 5′-GCCTGCAGTGCCCATTATG-3′;

ANXA2: 5′-CCTGCTCAGTATGACGCTTCTG-3′ and 5′-ACCATCAGCTTGCGGAAGTCAC-3′;

CCL2: 5′-AAGGGCTCGCTCAGCCAGATGC-3′ and 5′-GGAATCCTGAACCCACTTCTGC-3′;

tPA: 5′-CCTGCGGCCTGAGACAGTACAG-3′ and 5′-CGGAAACCTCTCCTGGAAGCAG-3′;

IL-6: 5′-CCAGAGCTGTGCAGATGAGTAC-3′ and 5′-CAGGCTGGACTGCAGGAACTCC-3′;

GAPDH: 5′-AGGGCTGCTTTTAACTCTGGT-3′ and 5′-CCCCACTTGATTTTGGAGGGA-3′.

### Cell transfection

Cells were grown to confluency on six-well plate and transfected with ON-TARGET smart pool siRNAs targeted to knock-down PAR1 (L-005094-00), PAR2 (L-005095-00), PAR3 (L-005491-00), PAR4 (L-005492-02) or FAK (L-003164-00), or a control non-targeting pool siRNA (Dharmacon; Lafayette, CO, USA) using DharmaFECT (Dharmacon). siRNA (25 nM/per well) and DharmaFECT (5 ul/well) were premixed in antibiotics- and serum-free DMEM/F12 medium for 20 min before application to the cells. After 24 h of transfection, the DharmaFECT-containing medium was replaced with DMEM medium containing 2% FBS. The transfection efficiency was more than 60%.

### Statistical Analysis

Statistical analysis was performed using the GraphPad Prism 6 (GraphPad Software Inc., San Diego, CA, USA). The values are presented as mean ± SEM. Statistical differences between the experimental group and the control groups were assessed using Student’s *t*-test. Statistical comparisons between more than two groups were performed using a one-way analysis of variance with a Bonferroni post-hoc test. The difference was considered significant if the *p*-value was <0.05.

## Results

### Patient characteristics and microarray

The demographics of the patients recruited into this study are presented in Tables 1 and 2. Pfirrmann grades I/II was used as the control/normal group, and grades III to V were used as the degenerative group. Degenerative grading was determined by a radiologist who independently evaluated the MRI according to the classification scale^36^. Disc pathology (contained and non-contained) were also analyzed by MRI. Contained disc herniation included disc protrusion, while non-contained disc herniation included disc extrusion or sequestration. In total, eighty-seven disc samples with Pfirrmann grade I/II (n = 10), grade III (n = 40), grade IV (n = 28) and grade V (n = 9) were collected. Typical MRI images of patients with Pfirrmann grades II-V are shown in Fig. 1A. The patient characteristics, including age (range 25–85 years), gender, surgical level, and disc pathology are listed in Table 2. The mean ages in years for each Pfirrmann grade were as follows: II, 48.7 ± 10.4; III, 52.1 ± 12.6; IV, 60.7 ± 10.7; V, 69.2 ± 11.7. There was a significant difference between age distribution and Pfirrmann grade (*p* < 0.001), but no significant difference in gender or contained vs. non-contained disc groups among the different Pfirrmann grades (p = 0.96 and 0.97). Furthermore, there was a high correlation (*p* < 0.001) between the Boos scores and the Pfirrmann grade (Fig. 1B). The NP cell numbers significantly decreased with the increasing IVD degenerative process (p = 0.001, Fig. 1C); specifically, the number of NP cartilage-like cells were 14.4 ± 4.99 (Pfirrmann grade II), 11.35 ± 4.8 (Pfirrmann grade III), 9.14 ± 4.47 (Pfirrmann grade IV), and 6.33 ± 3.16 (Pfirrmann grade V). To elucidate the possible mechanism underlying IVD degeneration and the decrease in NP cell number, we used a microarray approach to analyze gene expression of cell cultures from the different grades of NP and AF tissues. Since a decline of cellularity with clinical grade and the IVD exhibit a very limited capability for cell proliferation for self-repair^44^, we used a non-enzymatic method for isolating IVD cells, which preserves the phenotype of nucleus pulposus cells^39^, and approached for a microarray analysis. As shown in Fig. 1D, genes altered in NP and AF cells from each grade revealed a core of 172 genes that were changed 2-fold comparing AF with NP. Use of a KEGG pathway analysis of biological function with a strict p value cutoff of *p* < 0.01 resulted in the identification of eleven pathways (Fig. 1E). Because of IVD degeneration-caused back pain has been linked to ageing, excessive manual labour and genetic factors^45^, several previous clinical reports were focused on discovering the mechanisms of IVD cell death, cell cycle, DNA replication or DNA repair. However, there are other underlying events, such as injury of NP results in recruitment of immune cells to the disc which promotes low back pain^46^. From this perspective, in the present study, we would like to explore novel biomarkers for chemoattraction of immune cells. Therefore, we elected to exclude pathways involved in the modification of cell cycle, DNA function, reproduction, and cancer signaling, but instead we focused on the coagulation and cytokine pathways. We hypothesized that coagulation factors modulate the inflammatory mediators in the degenerative microenvironment of IVD.

### Thrombin effectively modulates cytokine and chemokine expression in NP cells

**Figure 2:**
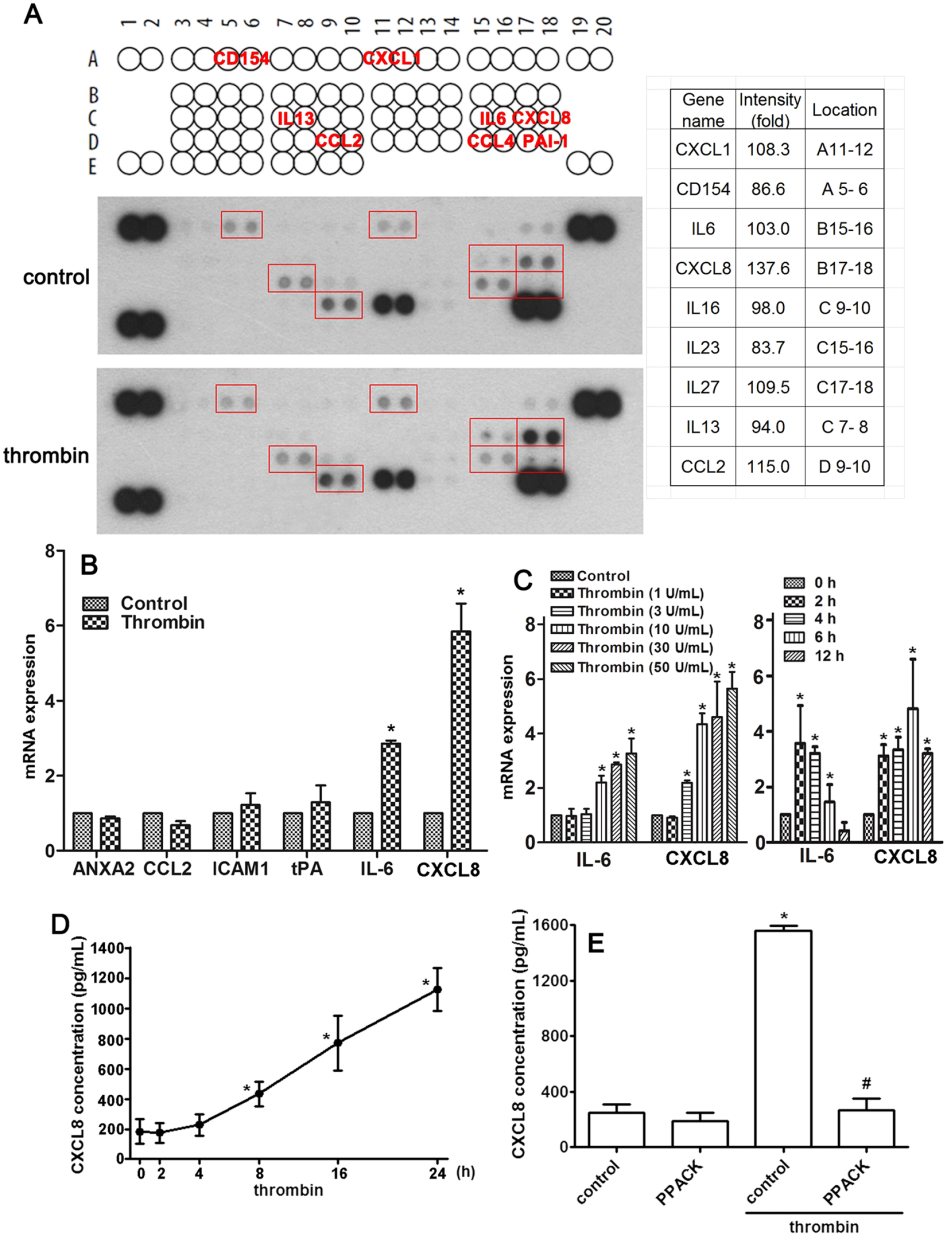
Regulatory effects of thrombin on cytokine and chemokine expressions in NP cells. (**A**) After treatment with thrombin, or an equal volume of vehicle, for 24 h, the supernatants from NP cell cultures were collected and applied to each of two separate cytokine/chemokine array membranes. The locations of cytokines and chemokines detected on the membrane are shown in the upper panel, and the quantitative data is shown in the right-hand panel. (**B**) Administration with thrombin (10 U/mL) to NP cells for 6 h, and the effect on mRNA of the indicated genes was determined using real time-PCR. (**C**) NP cells were treated with various concentrations of thrombin for 6 h, or 10 U/mL thrombin for the indicated time periods (2, 4, 8, 16 or 24 h). (**D**) Supernatants were collected for ELISA measurement of CXCL8. Each time point represents the mean ± S.E.M. from at least three independent experiments performed in duplicate. (**E**) NP cells were incubated with PPACK (1 μM) for 30 min followed by treatment with thrombin (10 U/mL) for 24 h, and CXCL8 protein levels were determined by ELISA. Each bar represents the mean ± S.E.M. from four independent experiments performed in duplicate. *p < 0.05 compared with the vehicle control group. ^#^ *p* < 0.05 compared with the thrombin-treated group.

**Figure 3:**
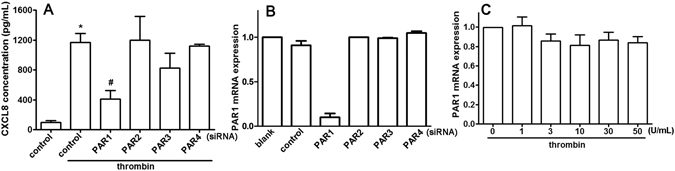
Thrombin-enhanced CXCL8 expression through PAR1 receptor. (**A**) NP cells were transfected with siRNA against PAR1, PAR2, PAR3, or PAR4, or the non-targeting control, for 24 h followed by treatment with thrombin (10 U/mL) for another 24 h, and then CXCL8 protein levels were determined by ELISA. Each bar represents the mean ± S.E.M. from at least three independent experiments performed in duplicate. (**B**) NP cells were transfected with siRNA against PAR1, PAR2, PAR3, or PAR4 for 24 h. (**C**) NP cells were treated with various concentrations of thrombin for 4–6 h. The gene expression of PAR1 receptor was determined by real time-PCR. Each bar represents the mean ± S.E.M. from three independent experiments performed in duplicate. *p < 0.05 compared with the control group. ^#^ *p* < 0.05 compared with the thrombin-treated group.

In order to validate the microarray observations, we performed a cytokine/chemokine array to examine the levels of expression of cytokine/chemokines after the addition of thrombin, the most important coagulation factor, to NP cells. As shown in Fig. 2A, thrombin increased the levels of a variety of inflammatory mediators expressed in NP cells culture supernatants, including CXCL1, IL-6, CXCL8, IL-27 and CCL2, whereas thrombin slightly decreased CD154, IL-16, IL-23 and IL-13 (right-hand table, Fig. 2A). The microarray data of CXCL1, IL-6, CXCL8, IL-27, CCL2 and CXCL8 in the different grades of NP tissue were shown in Table 4. Important to this study, the levels of CXCL8 were increased to the largest extent by 24 h thrombin stimulation compared to the vehicle control group. To further validate that thrombin enhances cytokines/chemokines and pro-inflammatory mediators expression, we treated NP cells with recombinant thrombin and analyzed the mRNA expression of inflammation related molecules. Treatment with thrombin significantly increased the expression of IL-6 and CXCL8, but not the levels of other pro-inflammatory mediators including ANXA2, CCL2, ICAM1 and tPA (Fig. 2B). Moreover, treatment with thrombin upregulated IL-6 and CXCL8 mRNA expression in a time- and dose-dependent manner (Fig. 2C). Treatment with thrombin also increased CXCL8 secretion as measured by ELISA assay (Fig. 2D). To further confirm this up-regulation of CXCL8 by thrombin receptor in NP cells, we used a thrombin inhibitor PPACK, which has been previously demonstrated to be efficacious in limiting thrombin-induced platelet activation^47, 48^. As shown in Fig. 2E, we found that PPACK completely inhibited thrombin-induced CXCL8 production. To further determine which PAR receptor was required for thrombin-induced CXCL8 expression, NP cells were transfected with siRNAs against PAR1, PAR2, PAR3, or PAR4 receptors and stimulated with thrombin. As shown in Fig. 3A, the thrombin-mediated induction of CXCL8 expression was abolished by the PAR1 receptor siRNA but not by PAR2, PAR3, or PAR4 siRNAs. In addition, these siRNAs specifically knockdowned the target genes more than 70%, but not crossed to other subtypes (Fig. 3B). Moreover, thrombin did not affect PAR1 expression itself in NP cells (Fig. 3C). Taken together, these findings demonstrate that the induction of CXCL8 by thrombin depends on PAR1 receptor in NP cells.

**Table 4:** The microarray data of CXCL1, IL-6, CXCL8, IL-27, CCL2 and CXCL8 in the different grades of NP tissues.

Gene Symbol	Fold change						Flags			Description
	(Grade 2 vs Grade 3)		(Grade 3 vs Grade 4 + 5)		(Grade 2 vs Grade 4 + 5)		Grade 2	Grade 3	Grade 4 + 5	
	Fold change	Regulation	Fold change	Regulation	Fold change	Regulation				
IL6	1.399	up	3.348	up	4.683	up	Detected	Detected	Detected	Homo sapiens interleukin 6 (interferon, beta 2) (IL6), mRNA [NM_000600]
IL27	1.508	up	1.366	up	2.060	up	Detected	Detected	Detected	Homo sapiens interleukin 27 (IL27), mRNA [NM_145659]
CCL2	1.996	up	2.914	up	5.815	up	Detected	Detected	Detected	Homo sapiens chemokine (C-C motif) ligand 2 (CCL2), mRNA [NM_002982]
CXCL8	6.629	up	1.792	up	11.878	up	Detected	Detected	Detected	Homo sapiens interleukin 8 (IL8), mRNA [NM_000584]
CXCL1	1.943	up	1.742	up	3.385	up	Detected	Detected	Detected	Homo sapiens chemokine (C-X-C motif) ligand 1 (melanoma growth stimulating activity, alpha) (CXCL1), transcript variant 1, mRNA [NM_001511]

### Thrombin-stimulated CXCL8 expression is mediated through activation of EGF receptor

**Figure 4:**
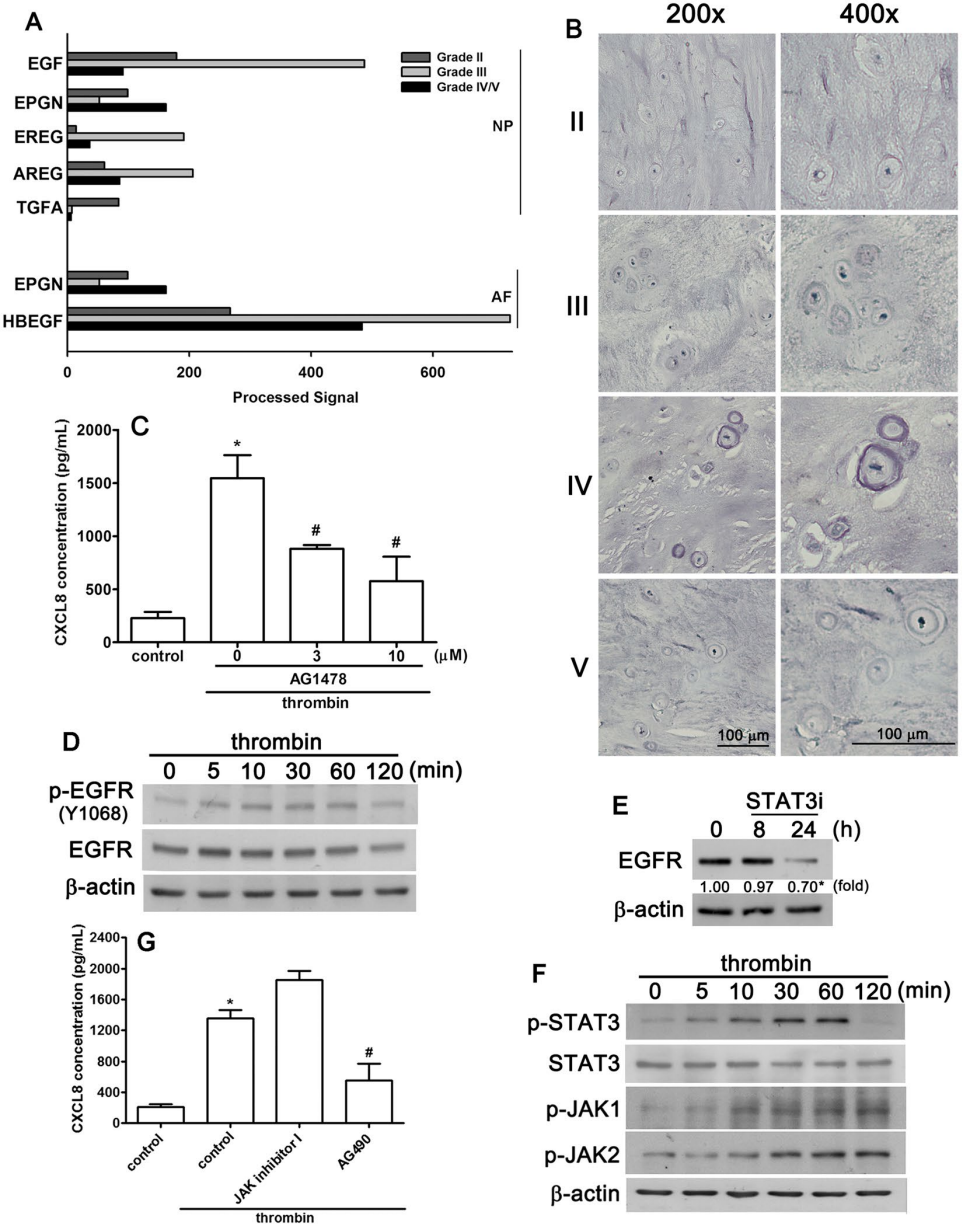
The EGF receptor is involved in thrombin-enhanced CXCL8 expression. (**A**) The mRNA expression of EGF receptor family members in patients with different MRI grades. (**B**) Representative immunohistochemical images of EGFR expression in paraffin-embedded sections at various MRI grades of human spinal disc specimens (three patients in each group). The extent of EGFR staining is slight in tissues with MRI grade III and V patients and strong in the tissue of grade IV patients. (**C**) NP cells were treated with AG1478 (3 or 10 μM) for 30 min following treatment with thrombin (10 U/mL) for 24 h; expression of CXCL8 was determined by ELISA. Each bar represents the mean ± S.E.M. from at least three independent experiments performed in duplicate. (**D**) NP cells were treated with thrombin for the indicated time periods, cell lysates were separated by SDS-PAGE and immunoblotted with either anti-phosphorylated EGFR (Tyr^1068^) antibody, anti EGFR antibody or anti β-actin antibody. (**E**) NP cells were treated with the STAT3 inhibitor for 8 or 24 h, and expression of the EGFR was determined by western blot. (**F**) NP cells were treated with thrombin for the indicated time periods and cell lysates were separated by SDS-PAGE and immunoblotted with anti-phosphorylated STAT3, anti-STAT3, anti-phosphorylated-JAK1, or anti-phosphorylated-JAK2 antibodies. Similar results were obtained from four independent experiments. (**G**) NP cells were pre-incubated with the JAK inhibitor 1 or AG490 for 30 min followed by treatment with thrombin (10 U/mL) for 24 h; CXCL8 protein levels were determined by ELISA. Each bar represents the mean ± S.E.M. from three independent experiments performed in duplicate. *p < 0.05 compared with the vehicle control group. ^#^ *p* < 0.05 compared with the thrombin-treated group.

In the microarray assay, inspection of the common genes altered in grade II to IV IVD degenerative specimens revealed five EGF receptor ligand genes that were increased two-fold in NP cells (EGF, EPGN, EREG, AREG and TGFA) but only two genes in AF cells (EPGN and HBEGF) (Fig. 4A). Next, we examined the expression of the EGF receptor in human degenerative IVD specimens using immuno-histochemical analysis. As shown in Fig. 4B, higher expression of the EGF receptor was found to be associated with a higher clinical pathological stage in the IVD degenerative specimens.

To establish that the EGF receptor mediates thrombin-induced CXCL8 expression, AG1478, a specific antagonist of the EGF receptor, was used. The thrombin-induced increase in CXCL8 expression was reduced by treatment with AG1478, the EGF receptor antagonist, in a concentration- dependent manner (Fig. 4C). Furthermore, the levels of the phosphorylated-EGF receptor (Tyr^1068^) were significantly increased in response to thrombin treatment (Fig. 4D). Phosphorylation of the EGFR achieved a maximum at 30–60 min and was sustained up to 120 min after thrombin stimulation. These results suggest that the stimulatory effects of thrombin on CXCL8 were mediated through the EGF receptor in NP cells.

We further investigated the signaling pathway involved in the EGFR-mediated stimulation of CXCL8 expression by thrombin. Surprisingly, administration with a STAT3 inhibitor for 24 h reduced EGF receptor expression (Fig. 4E). As shown in Fig. 3F, stimulation of NP cells with thrombin also resulted in the time-dependent phosphorylation of STAT3 without any effect on STAT3 protein levels. The phosphorylation of STAT3 reached a maximum 30–60 min after thrombin stimulation. In addition, the phosphorylation of both JAK1 and JAK2, which lie upstream of STAT3, were also increased in response to thrombin stimulation (Fig. 4F). Thrombin-enhanced CXCL8 expression was significantly reduced by treatment with AG490, a selective JAK2 inhibitor (Fig. 4G).

**Figure 5:**
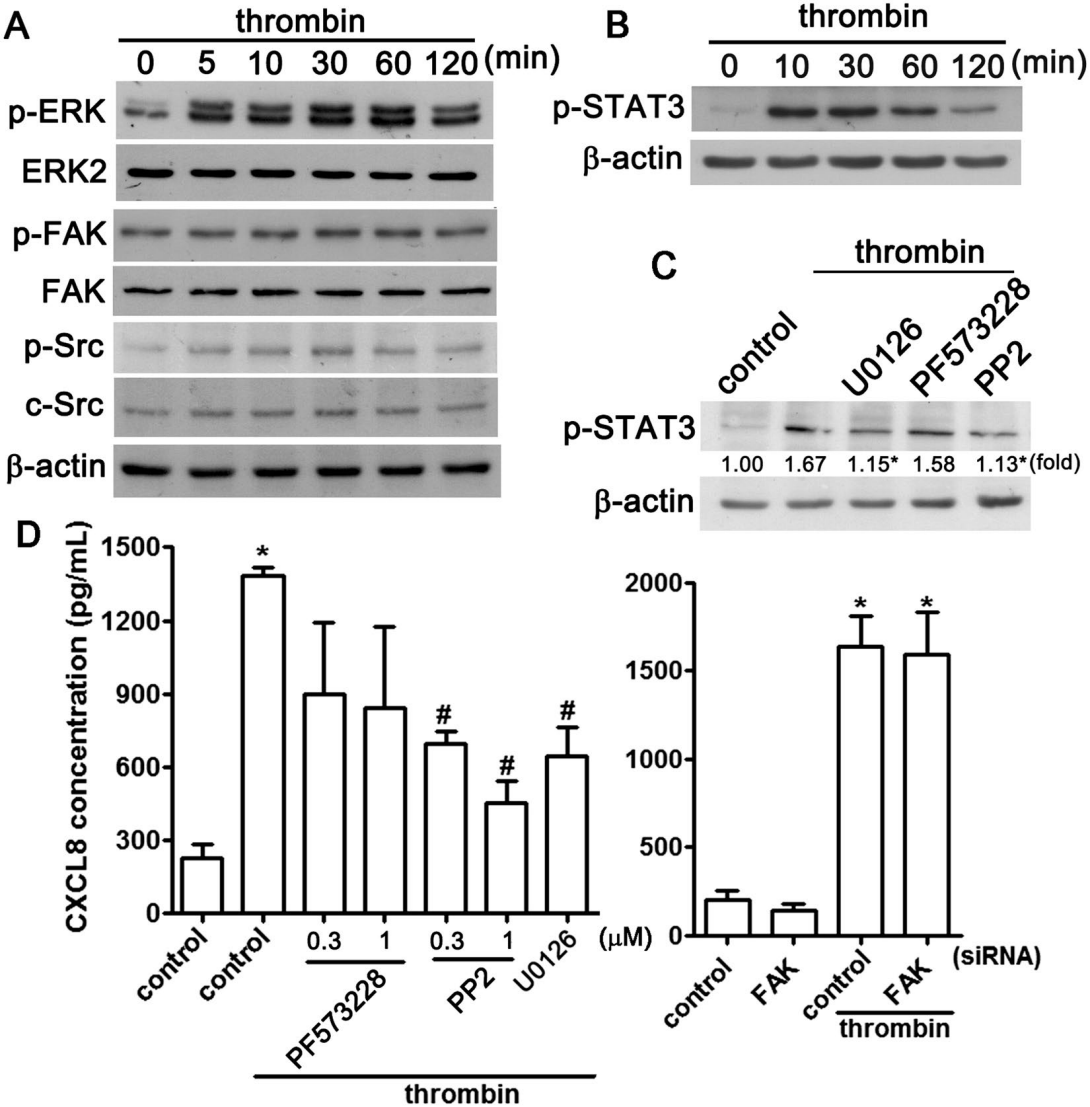
ERK and c-Src modulate thrombin-induced STAT3 activation in NP cells. (**A**) NP cells were treated with thrombin for the indicated periods (5, 10, 30, 60, or 120 min), cell lysates were separated by SDS-PAGE and immunoblotted with anti-phosphorylated-ERK, anti-phosphorylated-FAK or anti-phosphorylated-Src. (**B**) NP cells were treated with thrombin for indicated periods (10, 30, 60 or 120 min), cell lysates were separated by SDS-PAGE and immunoblotted with phosphorylated-STAT3. (**C**) NP cells were treated with U0126 (1 μM), PF573228 (1 μM), or PP2 (0.3 or 1 μM) for 30 min followed by treatment with thrombin for a further 60 min, and the levels of phosphorylated-STAT3 were determined by western blotting. Similar results were obtained from four independent experiments. (**D**) NP cells were treated with PF573228 (0.3 or 1 μM), PP2 (0.3 or 1 μM), or U0126 (1 μM) for 30 min followed by treatment with thrombin (10 U/mL) for 24 h (left-hand panel) and the levels of CXCL8 in the culture supernatant were measured by ELISA. NP cells were transfected with siRNA against FAK or non-targeting control for 24 h and were either left unstimulated or were stimulated with thrombin for 24 h before measurement of the CXCL8 levels in the culture supernatant (right-hand panel). Each bar represents the mean ± S.E.M. from at least three independent experiments performed in duplicate. *p < 0.05 compared with the control group. ^#^ *p* < 0.05 compared with the thrombin-treated group.

We then explored other signaling pathways that could also be involved in the up-regulation of CXCL8 after thrombin stimulation. As shown in Fig. 5A, treatment of NP cells with thrombin resulted in the time-dependent phosphorylation of ERK, which was initiated at 5 min, achieved a maximum at 30–60 min, and was sustained for up to 120 min after thrombin stimulation. In addition, thrombin also mildly induced FAK and Src phosphorylation in NP cells (Fig. 5A). As shown in Fig. 5B, stimulation with thrombin also increased STAT3 phosphorylation and this enhanced STAT3 activation was antagonized by inhibitors of Src (PP2) and ERK (U0126) but not of FAK (PF573228) (Fig. 5C). The same inhibitors were used to further confirm the signaling pathways that mediate thrombin-induced CXCL8 expression; the thrombin-induced increase in CXCL8 expression was markedly antagonized by treatment with PP2 and U0126 but not PF573228 (left-hand panel, Fig. 5D). In parallel, a siRNA against FAK did not affect thrombin-enhanced CXCL8 expression (right-hand panel, Fig. 5D). Taken together, these results suggest that thrombin stimulates ERK, c-Src, and STAT3 activation, and activation of the EGFR pathway is required for the up-regulation of CXCL8 in NP cells.

### Involvement of Akt and GSK3α/β signaling pathways in EGF receptor-mediated CXCL8 up-regulation

**Figure 6:**
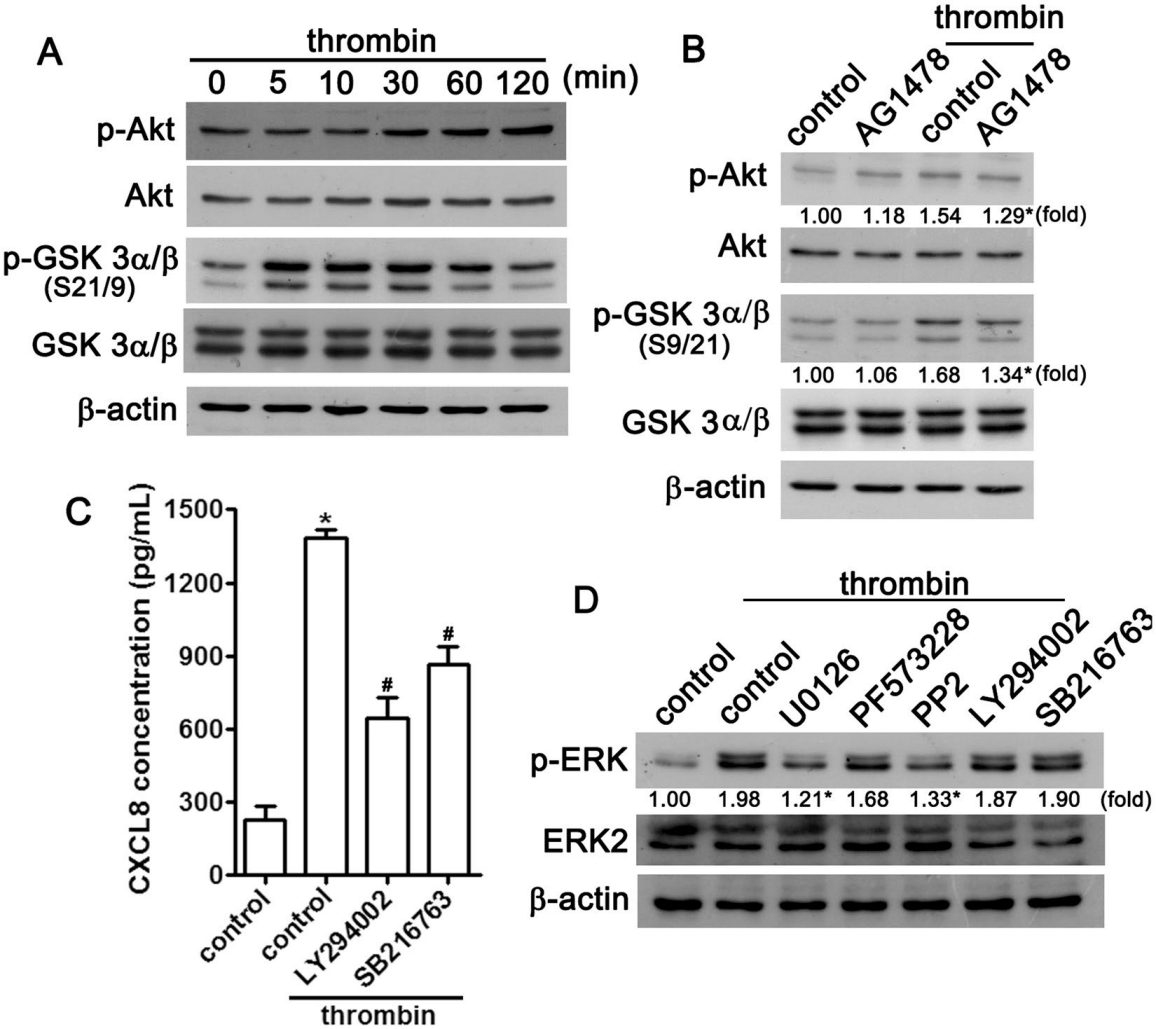
The Akt and GSK3α/β signaling pathways are involved in thrombin-enhanced EGFR activation in NP cells. (**A**) NP cells were treated with thrombin for indicated periods, cell lysates were separated by SDS-PAGE and immunoblotted with anti-phosphorylated-Akt and anti-phosphorylated-GSK3α/β. (**B**) NP cells were treated with AG1478 (3 μM) for 30 min followed by treatment with thrombin for 30 min, and the levels of phosphorylated-Akt, Akt, phosphorylated GSK3α/β, and GSK3α/β were determined by western blotting. Similar results were obtained from four independent experiments. (**C**) NP cells were treated with LY294002 (10 μM), or SB216763 (12.5 μM) for 30 min followed by treatment with thrombin (10 U/mL) for 24 h. The production of CXCL8 was determined by ELISA. Each bar represents the mean ± S.E.M. from at least three independent experiments performed in duplicate. *p < 0.05 compared with the control group. ^#^ *p* < 0.05 compared with the thrombin-treated group. (**D**) NP cells were treated with U0126 (1 μM), PF573228 (1 μM), PP2 (1 μM), LY294002 (10 μM), or SB216763 (12.5 μM) for 30 min followed by treatment with thrombin for 60 min, and phosphorylated-ERK and ERK2 levels were determined by western blot. Similar results were obtained from three independent experiments.

We then examined whether Akt and GSK3α/β, which are downstream of the EGF receptor, are also important in leading to increased CXCL8 expression in response to thrombin. As shown in Fig. 6A, treatment of NP cells with thrombin resulted in the time-dependent phosphorylation of Akt and GSK3α/β. Dual phosphorylation of GSK3α/β at Ser21 and Ser9 reached a maximum at 30 min and was sustained up to 120 min after thrombin stimulation. Moreover, treatment with AG1478 attenuated the thrombin-induced phosphorylation of Akt and GSK3α/β (Fig. 6B). To further confirm that these signaling pathways mediated the thrombin-induced CXCL8 expression, inhibitors of Akt (LY294002) and GSK3 (SB216763) were used. The thrombin-induced increase in CXCL8 expression was markedly antagonized by treatment with LY294002 and SB216763 (Fig. 6C). Additionally, thrombin-enhanced ERK phosphorylation was reduced by treatment with ERK (U0126) and Src (PP2) inhibitors but not FAK, Akt, and GSK3 inhibitors (Fig. 6D). Taken together, these data provide evidence that the downstream molecules Akt and GSK3α/β are involved in thrombin-induced EGFR activation and CXCL8 production in NP cells.

## Discussion

There is no curative strategy for degenerative disc disease (DDD) and the molecular mechanisms controlling IVD degeneration and accelerated cell death are not well understood. Given this lack of knowledge, the fact that there are no known biomarkers is hardly surprising. A potential therapeutic target that may offer the opportunity for medical intervention to the degenerating disc is therefore a widely sought goal. A previous microarray analysis of patients with IVD degeneration indicated that EGF expression in NP cells might play a key role in inflammation-associated IVD degeneration^49, 50^. In addition, a previous report also showed that EGF is expressed and produced by NP cells, both *in vivo* and in 3D culture, and this expression was significantly increased after inflammatory stimulation^51^. A recent study has also reported that periodic mechanical stress stimulates the phosphorylation of the EGFR in NP cells^52^. Importantly, the levels of EGF and CXCL8 correlated with pain and clinical parameters, including assessment of tear stability and tear production in patients with severe dry-eye disease. There are many transmembrane growth factor precursors that have been described as ligands for the EGFR, including EGF, heparin-binding EGF-like growth factor (HBEGF), amphiregulin (AREG), transforming growth factor-α (TGFA), betacellulin (BTC), epiregulin (EREG) and epigen (EPGN)^53^. In the microarray assay performed here, these EGFR binding ligands were up-regulated to a greater degree in NP cells than in AF cells (Fig. 3A). In addition, an analysis of patient IVD specimens showed that EGFR expression was positively correlated with the grade of tissue degeneration (Fig. 3B). In addition, thrombin enhanced EGFR phosphorylation, and treatment with an EGFR inhibitor effectively reduced thrombin-induced CXCL8 expression (Fig. 3C). Our results also support previous reports that thrombin stimulates pathological and biological functions through EGFR activation^54, 55^. Our results provide a clue that EGFR might be a potential biological target in IVD diagnosis and for development of a therapeutic drug.

EGFR is a receptor tyrosine kinase (RTK) which can be transactivated by G protein-coupled receptors (GPCRs) through an EGF ligand-independent manner, which suggested to be regulated through intracellular signaling pathways^56^. Daub *et al*., 1996 firstly provided the evidence that transactivation of EGFR was following the addition of the GPCR agonists such as thrombin^57^, which activation was blocked by the EGFR kinase inhibitor AG1478^57^. This regulatory effect could be classified into two major mechanisms, including through the triple membrane passing signal (TMPS) pathway and through the EGF-like ligands-independent pathway^56, 58, 59^. The EGFR transactivation through TMPS pathway controlled by the activation of membrane-bound matrix metalloproteases has been reported in many cell types following activation by various agonists like thrombin^60^. On the other hand, the ligand-independent transactivation of EGFR is phosphorylated EGFR in its cytosolic domain by the activation of intracellular protein tyrosine kinases like Src family proteins^58, 59^. It has also been reported that Src activation plays a permissive role for PAR1-mediated transactivation of EGFR in colon cancer cell growth^61^. Previous report also found that FAK plays an essential role for the transactivation of EGFR^62^. Present study supported previous reports that phosphorylation of EGFR is a critical for thrombin-induced CXCL8 overexpression, and the EGFR kinase inhibitor could attenuate the enhancement effect. Our results also found that Src and FAK activation are downstream of thrombin stimulation. However, the key kinases directly modulate the transactivation of EGFR in NP cells need further investigation.

IVD degeneration is associated with inflammation, which can become chronic^63^. Beyond the potential application to new therapeutics, the novel hypotheses and mechanistic studies portrayed here have the potential to describe how the pathophysiologic state in IVD is modulated. Thrombin is generated following joint inflammation in osteoarthritis patients^64^ and following the abnormal proliferation of synovial cells in rheumatoid arthritis^65^. Importantly, the use of a thrombin antagonist has been proposed as a novel therapeutic strategy for the suppression of inflammation and degeneration in nervous system injury^66–68^. In this regards, a means of attenuating the untoward effects of thrombin is a goal of current research efforts. Patients with radicular pain caused by lumbar spinal stenosis, who received epidural injections of the anti-IL-6R monoclonal antibody tocilizumab, showed improvement in lower back and leg pain. Moreover, epidural administration of the TNF inhibitor etanercept to the spinal nerve provided pain relief in patients experiencing radicular pain arising as a result of lumbar spinal stenosis^69^. In a randomized controlled study, the anti-TNF antibody adalimumab^70^ and infliximab^71^ showed a marked improvement in leg pain and a reduction in the number of patients with IVD-herniation-induced sciatica. Furthermore, the combination of anti-IL-8/CXCL8 antibodies enhances the effect of an anti-TNFα antibody on mechanical hyperalgesia in both the autologous IVD autograft model and the spinal nerve ligation model^72^.

Higher levels of the inflammatory mediator C-reactive protein (CRP) have been found in patients with sciatic neuropathy versus healthy controls^73^. Previous studies have suggested that NP cells may release many mediators that contribute to the pain experience. Inflammatory agents that might be released from NP cells could affect the dorsal root ganglion to enhance the primary afferent excitation of nociception^74^. In addition, NP cells applied onto the dorsal nerve roots enhance the spinal C-fiber response and macrophage activation^75^. Importantly, the release of CXCL8 from the NP plays a critical role in IVD degeneration and immune cell infiltration^35^. Clinically, over-expression of the chemokine CCL3 has been shown to be positively correlated with the grade of tissue degeneration by recruiting macrophages^76^. Clinical observations have also shown that serum levels of CXCL8 are associated with long-lasting pain in patients with disc herniation^77^ and increased levels of CXCL8 in the cerebrospinal fluid are also correlated with disc herniation^78^ and worsening of fibromyalgia-related pain severity^79^. Moreover, degenerated and herniated human intervertebral discs may secrete high levels of CXCL8^80^. It has also been reported that intra-plantar CXCL8 injection induces a persistent hypersensitivity^81^, which can be blocked using a CXCL8 receptor antagonist^34^.

A previous study has reported that ERK1/2 phosphorylation in NP cells has been observed in degenerated human intervertebral disc^82^. Moreover, thrombin also enhances CXCL8 expression through ERK activation^83^. It has also been demonstrated that transcriptional activation of the phosphoinositide 3 (PI3)-kinase/Akt pathway is involved in lumbar disc degeneration^84^ and thrombin activates the PI3 kinase/Akt signaling in neural retina^85^. A recent report, using bioinformatics, also indicated that ERK, PI3 kinase/Akt, and EGFR (ErbB) signaling pathways are potentially involved in intervertebral disc degeneration^50^. Beyond these, Src and FAK have been reported as being key early signaling molecules that mediate neurite growth in response to growth factor stimulation^86^ and thrombin has also been reported to induce c-Src activation^87^.

In conclusion, using standard molecular biological tools we have identified CXCL8 as a critical cytokine/chemokine important to IVD cells. Using a microarray of human tissues and *in vitro* stimulation of healthy NP cells, we confirmed a role for thrombin and the EGF receptor signaling pathways in the onset of IVD. This study suggests that targeting the EGFR pathway may be of crucial importance in the preventive treatment of disc degeneration that leads to chronic low back pain. The results of this study may also contribute to the development of post-operation treatments and/or a diagnosis for the IVD degenerative disease.
